# The value of case reports in democratising evidence from resource-limited settings: results of an exploratory survey

**DOI:** 10.1186/s12961-020-00592-y

**Published:** 2020-07-20

**Authors:** Marta A. Balinska, Richard A. Watts

**Affiliations:** 1 Vienna Evaluation Unit, Médecins Sans Frontières Austria, Taborstrasse 10, 1020 Vienna, Austria; 2 Norwich Medical School, University of East Anglia, Norwich, United Kingdom

**Keywords:** Case reports, Case management, Patient-centred care, Capacity building, Teaching, Clinical ethics, Low- and middle-income countries, Humanitarian and resource-limited settings

## Abstract

**Background:**

Following a knowledge management analysis, Médecins Sans Frontières (MSF) – a medical humanitarian non-governmental organisation (NGO) – identified significant loss of medical knowledge from the field, owing primarily to the absence of a platform on which to share clinical lessons learned in humanitarian and resource-limited settings (HRLS). Wishing to address these missed opportunities to retain important scientific and pragmatic knowledge, the NGO has begun to actively encourage its clinicians to publish case reports/series that bring new and/or practical insights of benefit to patients and population groups. In parallel, we wished to obtain a clearer understanding of how case reports (CRs)/series can best play their role as ‘first-line evidence’ from HRLS, especially in areas suffering from a significant lack of data.

**Methods:**

We developed a survey with closed and open questions on ‘The value of CRs from HRLS’ to explore primarily (1) the reasons why this form of evidence from HRLS is often lacking, (2) what makes a case report/series worth sharing with the wider global health community, and (3) how we can ensure that published case reports/series reach their target audience.

**Results:**

Over a 6-month period, 1115 health professionals responded to the survey. Participants included clinicians and public health specialists from all over the world, with a majority based in Africa. The main reason cited for the dearth of CRs from HRLS was that practitioners are simply not writing and/or submitting reports (as versus having their papers rejected) due mainly to (1) a lack of skills and (2) time constraints. A large majority of respondents felt the CRs are a valuable tool for HRLS given their ability to discuss how cases are managed with rudimentary means as well as to draw attention to emerging or underestimated public health problems and neglected populations.

**Conclusion:**

We conclude that the clinical knowledge gained in resource-challenged settings is being underutilised in the interest of patients and global health. Consequently, clinicians in HRLS need greater access to basic training in scientific investigation and writing in addition to awareness as to the potential value of sharing their clinical experience with a view to broadening evidence production from high-income to low-income settings.

## Background

Médecins Sans Frontières (MSF) is a medical humanitarian non-governmental organisation (NGO) that has been delivering aid in emergency and resource-limited settings for almost half a century. Currently, it has over 60,000 staff scattered across the globe. As part of a strategic project looking at how we manage our medical knowledge, a needs assessment revealed loss of valuable clinical experience gained in difficult field conditions due to the absence of a platform on which clinicians can share the lessons learned. This loss is further impacted by significant staff turnover and was deemed to primarily affect the quality of care and patient safety as well as to lead to redundancies in medical investigation [1].

At MSF, emphasis has traditionally been placed on operational research with less attention to individual patient care. Every year, MSF-affiliated authors publish around 200 articles in medical journals. In 2017, 63% of these were peer-reviewed research articles and the rest were commentaries or advocacy pieces. Despite MSF’s policy to publish in open access journals, 24% of these papers came out in paid subscription journals. The most frequent topics addressed were HIV and tuberculosis, whereas other core topics, such as antibiotic resistance or sexual violence, were under-represented. Over the last decade, there have been very few published case reports (CRs)^1^ – probably fewer than 20 [2].

Beyond MSF, it has been well established that fewer scientific papers are published from ‘third world’ countries compared to ‘first world’ countries even in areas such as tropical medicine, where the former bear the greatest burden of disease. In 2004–2006, a series of articles took a closer look at this gap and suggested that second- and third-world authors fear rejection, have insufficient writing skills, and overall lack incentive to engage in science [2–6]. Since then, other studies have shown also that scientists from developing countries are poorly represented on editorial boards of prestigious journals and that there is a higher peer rejection rate for papers submitted by authors in middle- and low-income countries [7–11].

But what does this mean in practice? For one, it means that a clinician in a low-resource setting faced with a complex case will find published evidence from high-resource settings but not necessarily and not often applicable to their own context. Second, it means that valuable insights for clinical practice in poorly resourced areas are invisible because they never get published. Third, it points to non-English speakers/readers as being at the greatest disadvantage for accessing and contributing to the published literature. A further thought-provoking consideration is the idea that healthcare organisations whose staff engage in research experience overall improved performance. Several studies have tested this hypothesis in high-resource settings [12, 13] but, to our knowledge, none have been conducted in low-income contexts.

The CR has fallen somewhat out of fashion in the era of evidence-based medicine because of its intrinsic anecdotal quality – observations at the bedside on a single patient or even a group of patients (but not randomly selected) quite obviously do not provide statistical evidence. However, that is not a reason to dismiss patient-centred observational studies as a source of evidence altogether. To begin with, they have played a key role through the millennia of medical history as a means for signalling new illnesses and/or treatments and outcomes but also, crucially, for teaching new generations of healthcare practitioners. Further, CRs capture information that cannot be detected by statistical studies, such as rare events, as well as in-depth patient characteristics and the ‘art’ of clinical practice. Finally but importantly, rigorous CRs can be written by a range of clinicians without requiring specialised training in medical statistics, research teams or research budgets. This means that they give a ‘voice’ to practitioners not working in high-resource settings where conducting research is common. Thus, CRs can be an excellent channel for low-resource settings to communicate about the kind of health conditions they are seeing and, as we all know, the CR is almost always the first step of medical investigation and the prerequisite for larger, statistical studies testing hypotheses [14–17].

In the light of these findings, we wondered (1) are CRs from humanitarian and resource-limited settings (HRLS) being rejected, published in local journals, or simply never written? (2) What is the impact and significance of this publication bias? (3) Can CRs – which do not require expensive research budgets or statistical skills – be used to generate data and identify when there is a need for more evidence, larger studies and/or contextualised clinical guidelines? And, more generally, how should we be writing CRs from HRLS so that they can be used for evidence-based guidance in medicine and public health?

## Methods

We conducted an online bilingual (English, French) survey on ‘The value of CRs from HRLS’ with open and closed questions from June through December 2018. On the survey web page, we briefly described the MSF initiative to encourage case reporting from the field and provide training and editorial support in view of publication in peer-reviewed international journals (the Clinical Case Reporting Initiative (CCRI) at MSF).

We relied on convenience sampling with several thousand invitations to participate sent through email lists of two MSF operational centres and Telemedicine database, the Global Health Network, *Oxford Medical Case Reports*, and the Health in Humanitarian Crises Centre of the London School of Hygiene & Tropical Medicine. Given that our objective was to collect information, ideas, and opinions and not to test a hypothesis as such, we did not engage in power sampling or design the study to compare types of respondents. The questionnaire was developed on the basis of an earlier (unpublished) needs assessment survey with field practitioners about medical knowledge management at MSF as well as on the literature looking at advantages and limitations of CRs as a study type [14–17] and addressing specific issues typical for the field such as loss of patients to follow-up and lack of informed consent.

We analysed the data both from a quantitative perspective (for closed questions) and a qualitative perspective (for open questions), which we categorised according to larger groups.

Below, we provide descriptive statistics.

## Results

**Fig. 1:**
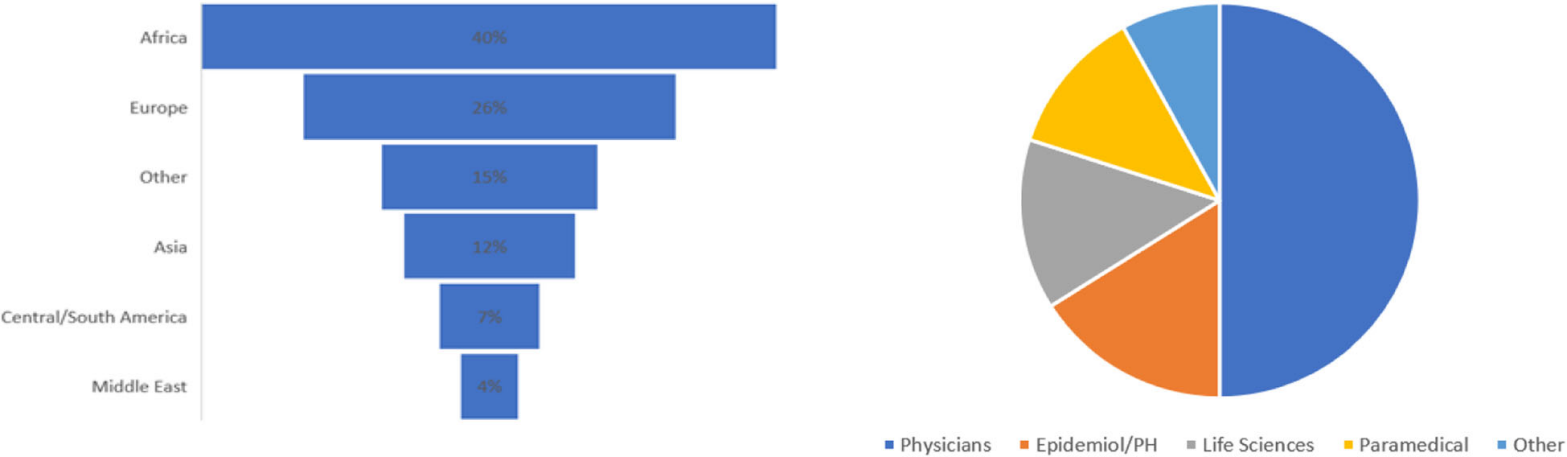
Geographical location and professional make-up of respondents

A total of 2368 individuals clicked on the survey link; 1115 answered some of the questions and 740 responded to all questions (including open questions). The geographical location and professional background of participants are shown in Fig. 1.

A large majority of respondents (65%) stated that CRs were directly relevant to their practice, teaching or research. Their strong points, as a form of medical evidence, was seen to be their capacity to detect emerging or unrecognised public health problems and to describe rare or novel events. Their weak points were the “*subjectivity*” of choice as to which cases get published and the lack of “*standardised*” and “*user-friendly*” reporting guidelines.^2^

When asked “*what makes a CR worth publishing?*”, respondents pointed, in the first place, to reports of public health significance but suggested many other valuable criteria by which to select reports of importance for the wider health community, as shown in Table 1.

**Table 1:** Criteria for publishing a case report/case series

Criterion	Highly important	Somewhat important	Not so important	NA
… a neglected public health issue	59%	21%	3%	17%
… an unexpected treatment outcome	55%	23%	4%	18%
… a rare presentation of a common condition	52%	27%	5%	16%
… a rare condition	51%	25%	7%	17%
… an unusual treatment protocol	49%	26%	6%	19%
… an issue of differential diagnosis	42%	32%	8%	18%
… an ethical dilemma	41%	30%	11%	18%
… case management of pedagogical value	39%	34%	9%	18%
… advocacy for patient groups	35%	31%	14%	20%

Other criteria making a case report ‘worth publishing’ that were cited, included (according to importance):

- If it illustrates how complex cases can be managed with very limited resources, with non-specialised/overextended staff, and/or in a sustainable way

- If it reports re/emerging diseases, new disease patterns

- If it contributes to improved quality of care, new models of treatment

- If other (statistical) evidence is lacking; if it can be used to justify larger studies

- If the condition is associated with cultural practices or ethnic groups

- If the condition is associated with high mortality

- If the case shows up success points or insufficiencies of the health system in place

- If the case is about the management of multiple morbidities

To the question of why seemingly few CRs are being published from HRLS, a majority (65%) felt that clinicians in these contexts are simply not submitting reports for publication. However, respondents also pointed out that high-profile international journals may be mostly geared to high-resource settings (51%) and/or that papers from low-resource settings may be rejected due to language issues (29%). Specific obstacles for field clinicians were identified as their insufficient skills in scientific writing (67%), their lack of awareness as to the potential value of sharing CRs (52%) and language barriers (31%). A few respondents thought that open access publication charges or subscription fees could also be influencing the gap in the literature.

Since patients in HRLS can be often lost to follow-up, meaning that informed consent may not have been secured for a case that is subsequently deemed worth publishing, participants were asked if they felt it was legitimate to go ahead and publish if all direct and indirect potential patient-identifiers (including author names) have been removed – 44% responded yes, 26% no, 17% were uncertain and 13% gave no answer.

Respondents were asked for their opinion about broadening the scope of CR authors beyond physicians. Those in favour (78%) stressed the importance of the clinical “*team*”, with a “*multidisciplinary*” and “*holistic*” approach, and that in many HRLS settings paramedics (clinical officers, nurses, etc.) – and not physicians – may be in charge. Other respondents suggested that all those who work in the health sciences (including social scientists) should have the skills to write a CR. Only 6% felt that authorship should be restricted to medical doctors, arguing that they have ultimate responsibility for patients, better writing skills and are more highly respected by their peers.

Respondents were favourable to including the patient’s perspective in terms of treatment acceptability, tolerance and adherence (77%); cultural and/or personal perception of illness including causality (70%) and subjective experience of symptoms/pain (60%).

The prime target audiences for CRs were seen to be local clinicians working in HRLS (53%), followed by the global health community (45%) and international clinicians working for NGOs and development agencies (45%). In terms of knowledge dissemination, it was felt that websites of organisations working in HRLS would be the best communication channel (60%) but also journal websites (48%) and professional social media (37%).

Respondents were asked to comment in any way they wished on the CCRI using free text. Most stated that it is a long overdue initiative, that it can bring about substantial change in clinical practice, is a great opportunity for field clinicians to engage in science and can lead – via the publication in a high-quality journal – to a valuable database on complex case management in HRLS. Further responses are shown in Table 2.

**Table 2:** Reponses to the open question for comments on the clinical case reporting initiative at MSF

“*Case reporting is of utmost importance. High powered advocacy must be raised to sensitize Africa especially...*”	
“*Case reporting is an essential but undervalued generator of evidence, for practice, policy, and bioethics.*”	
“*This is a very pertinent project and will give MSF scientific visibility on top of humanitarian visibility.*”	
“*This is really a good opportunity for physicians working in third world countries.*”	
“*This is a very good idea. I work in Africa. A great number of times I face situations that one obscure case report makes a lot of difference. Sometimes I see some rare but unpopular catastrophic situations and surmount them, and I think it would be great to have a forum where people like me can be heard. Most importantly, there are times I am [helped] by a case report written by someone who has been in my situation before. The online searches are usually negative, or I am asked to pay. The places I work cannot afford to pay me enough for online subscriptions in US dollars. Even when I am desperate and willing enough to pay.*”	
“*Make sure medical professionals in the field know that MSF is looking for and supporting case reporting. Offer help, both in content, time, and writing. I personally would need this help.*”	
“*Please let us know where and when is the next* [training venue in case report writing]*!*”	

## Discussion

The main limitation of our survey is its obvious potential for response bias – those more interested in CRs and patient-centred research are more likely to have participated and, consequently, may also have overrated the value of CRs as a form of medical evidence from HRLS. However, it clearly demonstrates that CRs are a recognised method of communicating the experience of educational and clinical value to a wide community and that there are currently significant barriers to the dissemination of such knowledge from HRLS. These barriers include insufficient skills and experience in writing CRs, together with potential financial cost, as other studies have suggested [3–11].

It has been shown that clinicians in the field would like detailed, user-friendly and contextualised reporting guidelines to help them report cases of interest. The open questions clearly indicated that what field clinicians would find most useful is a CCRI interactive web platform with tools, documents, webinars, discussion forums and spaces for communities of practice to share and discuss a variety of clinical and public health topics. Guidelines for writing CRs do exist and have been published (http://www.equator-network.org/reporting-guidelines/care/). However, publication from HRLS poses specific challenges: especially consent, as often follow-up may be limited or – in an emergency setting for example – inexistent. In addition, socio-political complexities may require complete anonymity both for the patient and the author or may bar publication of patient data altogether. Finally, aggregate data from case series may raise the question of data ownership, which can be tricky. We are actively addressing these issues as part of the project.

Further, our results reinforce the importance of patient-centred research, alongside operational research, for comprehending complex health conditions in developing countries and for improving the quality of care. CRs can play a significant role in identifying subjects that may be needed to link the knowledge gained in the field with policy and practice and inform clinical practice guidelines [18, 19]. This reflects a more recent realisation that, while CRs may be low on the evidence-based medicine pyramid, they have a key role to play as a foundation of medical observation and often spur on further (statistical) research [20, 21]. This is still truer for HRLS where we are dealing with multiple neglected health conditions and populations. As one specialist put it: “*Case reports and case series may be seen as the ‘lowest’ and ‘weakest’ level of evidence, but they often remain the ‘first-line of evidence’. This is where everything begins*” [22].

Going beyond the observation that relatively few CRs from HRLS are being published in international journals, this survey suggests that (1) clinical experience/knowledge from HRLS is being underutilised; (2) clinicians working in HRLS need access to basic training in scientific investigation and writing, and (3) there is a need and willingness in developing countries to come back to patient-centred approaches in addition to epidemiological research.

Awareness is growing that standards of care are set internationally to match resources in high-income countries [23]. The fact that these diagnostic and therapeutic resources may be totally absent in middle- and low-income countries has been largely overlooked. The CCRI is a step in the direction of democratising evidence production in order to give a voice to clinicians working in precarious situations and, ultimately, improve the standards of care in resource-challenged settings. Further research on how engaging field clinicians in science affects the quality of care in their healthcare organisation is warranted.

Although MSF is happy to be taking the lead on this initiative, we do not intend to limit it to MSF staff but hope to attract other health professionals and academics working in HRLS to join in our evidence-generating efforts, profit from and contribute to the training and tools we are developing.

## Conclusion

We conclude that clinical knowledge gained in resource-challenged settings is being underutilised in the interest of patients and global health knowledge. Specifically, there is a pressing need to broaden evidence production to include low-income settings and authors from developing countries. Our study clearly shows that clinicians in HRLS need greater access to basic training in scientific investigation and writing in addition to awareness as to the potential value of sharing their clinical experience.

The corresponding author invites anyone reading this article and interested in learning more about the CCRI to contact her directly.

## Data Availability

The datasets generated and/or analysed during the current study are available from the corresponding author on reasonable request.

## Abbreviations

CR: Case report
CCRI: Clinical Case Reporting Initiative
HRLS: Humanitarian and resource-limited setting
MSF: Médecins Sans Frontières (Doctors Without Borders)
NGO: Non-governmental organisation

## Footnotes

1 Throughout this article, the term ‘case report’ will refer both to case reports of one or two patients and to case series of more than two patients.

2 In fact, there do exist reporting guidelines for case reports available on the Equator Network: the CARE Guidelines (http://www.equator-network.org/reporting-guidelines/care/).
